# Women's Experiences of Recurrent UTIs: A Qualitative Study of Preventive Management and Decision Support Needs

**DOI:** 10.1111/hex.70890

**Published:** 2026-10-03

**Authors:** Catherine V. Hayes, Donna M. Lecky, Haroon Ahmed, Rebecca Cannings‐John, Adrian Edwards, Fiona Wood, Leigh Sanyaolu

**Affiliations:** ^1^ Primary Care and Interventions Unit UK Health Security Agency (UKHSA) Gloucester England UK; ^2^ Division of Population Medicine and PRIME Centre Wales Cardiff University Cardiff Wales UK; ^3^ Centre for Trials Research Cardiff University Cardiff Wales UK

**Keywords:** patient experience, prevention, primary care, qualitative research, recurrent urinary tract infection, shared decision‐making

## Abstract

**Introduction:**

Recurrent urinary tract infections (rUTIs) are common in women and significantly impact their daily lives. Prophylactic antibiotics are the primary preventive option for rUTIs when other options have been unsuccessful, but their use can increase the risk of antimicrobial resistance. This study aimed to explore women's experiences of rUTIs and the support needed to make decisions about prevention and management.

**Methods:**

Qualitative focus groups were conducted with 25 women with self‐reported rUTIs, discussing hypothetical vignettes related to rUTI management. Topic guides were co‐produced with women with lived experience and informed by the Theoretical Domains Framework (TDF). Data were analysed thematically.

**Results:**

Themes included: (1) Recurrent UTIs are a debilitating condition, (2) Current healthcare does not support women with rUTIs, (3) ‘Why is this happening?’ (4) Preventive options are frequently tried with varying success and (5) Greater support is needed for women with rUTIs. Overall, women experienced rUTIs as a chronic condition and believed it should be managed with greater continuity. Women experienced stigma attached to rUTI, exacerbated by repeated discussions about hygiene from healthcare professionals (HCPs). Women had concerns around antibiotic use but were reluctant to stop prophylaxis if it was effectively preventing rUTIs; a contingency plan to address potential recurrences could help. Women reported mixed success with non‐antibiotic strategies but were interested in trialling these with greater guidance from HCPs.

**Conclusion:**

Improved management of rUTI requires greater continuity and proactive discussions on prevention, including better support for discussions on risks and benefits to enhance shared decision‐making.

**Patient or Public Contribution:**

Four female public contributors with experience of rUTI were involved from study planning to completion. They helped co‐develop the topic guide vignettes, suggested potential recruitment routes and commented on the interpretation of themes in the context of their lived experience.

## 1 Introduction

Recurrent urinary tract infections (rUTIs) are defined as two or more acute urinary tract infections (UTIs) within 6 months, or three or more within a year [1]. They have an estimated UK prevalence of 6% for women [2]. rUTIs are a significant health concern, often resulting in frequent antibiotic exposure for patients who may receive antibiotics for acute episodes as well as for long‐term prevention [3]. This means patients with rUTI are at an increased risk of developing antimicrobial‐resistant (AMR) infections. A recent cross‐sectional analysis of Welsh primary care data found that 81% of women with rUTI had a urine culture in the previous year, of whom 40% and 57% had evidence of resistance to two prophylactic antibiotics, trimethoprim and amoxicillin, respectively [2]. This is concerning since AMR is a major global health threat, and patients who experience antibiotic‐resistant UTIs have worse health outcomes, a greater chance of hospitalisation and significant health service costs [4, 5].

There are several preventive strategies for rUTI, some of which have mixed evidence. Prophylactic antibiotics can be taken as a daily low dose or in response to an identified trigger, such as sexual activity [1]. The use of prophylactic antibiotics for 6–12 months to prevent UTI episodes reduces recurrence when compared to placebo [3]. However, the accompanying risks for AMR and side effects mean patients and clinicians have concerns about their use, with patients requesting more discussion about non‐antibiotic prevention [6]. Recent updates to the UK National Institute for Care Excellence (NICE) guidance indicate a move to non‐antibiotic options for prevention, including methenamine hippurate [1], a urinary antiseptic, that has been demonstrated to be non‐inferior to antibiotics [7]. NICE also recommends vaginal oestrogen for patients who are peri‐menopausal, currently menopausal or post‐menopausal. The incidence of UTIs increases with age, particularly in those over 65 years, with declining oestrogen believed to be an important factor [8]. However, this means that preventive options are not universally applicable across all patient groups, as the range of effective options differs according to menopausal status. Additionally, NICE advocates that all women with rUTIs are given advice around self‐management strategies. Increased fluid intake is associated with significantly fewer recurrences in women [9], and while cranberry, d‐Mannose and probiotics have inconsistent evidence of effectiveness [1], patients often report benefit [6]. Whilst hygiene measures such as urinating after sexual intercourse, direction of wiping after the toilet or avoidance of tight clothing are widely recommended, there is no robust clinical evidence of effectiveness [10].

Shared decision‐making (SDM) is a key component to support patients with prevention and management, and involves patients being aware of the benefits and risks of treatment options and exploring their own preferences [11]. Qualitative research helps to foreground women's voices and lived experiences to inform better individualised care. As well as experiencing significant quality of life impacts, patients often experience barriers to healthcare and negative, often dismissive interactions with health professionals [12, 13, 14, 15]. While qualitative evidence has provided insights into patient experiences, there is a lack of literature on the decision‐making experiences of women with rUTI around non‐antibiotic preventive options [6], which could help capture the nuanced personal experiences of women with this condition.

The aim of this study was to explore the experiences of women with rUTIs and how they manage their condition, including experiences of self‐directed and healthcare‐initiated preventive options. A key objective was to understand experiences of healthcare interactions in the context of prevention, and identify support needed for SDM about prevention and management.

## 2 Materials and Methods

### 2.1 Study Design

This qualitative study used semi‐structured focus groups supported by vignettes. Focus groups were stratified by menopausal status, as it impacts prevalence and treatment options, and shared experiences were more likely within these groups [16]. The Theoretical Domains Framework (TDF), used frequently in the context of UTIs [12, 17], helped guide the design of the topic guide and to map the themes of the analysis [18]. It was used more as a sensitising framework rather than as the primary interpretive lens; interpretation was guided chiefly by the data and research aims.

The topic guide (Supporting File S1) included vignettes, informed by previous literature and iteratively developed by the study team and public involvement and engagement group (PPIE). The vignettes (Figure 1) aimed to narratively explore stages of a patient's hypothetical journey with rUTI, allowing participants to discuss their thoughts within the context of the character's story, and share personal experiences if they felt comfortable doing so [19]. These represented realistic scenarios based on the clinical experience of the study team and the lived experience of the PPIE group. Based on PPIE input, five versions of the vignettes were developed to reflect the age groups, life stage, and menopausal status of focus group participants. A pilot of the vignettes was conducted in a mock focus group with the PPIE group to help refine the final versions. The Consolidated Criteria for Reporting Qualitative Research (COREQ) was used to report this study (Supporting File S2).

**Figure 1:**
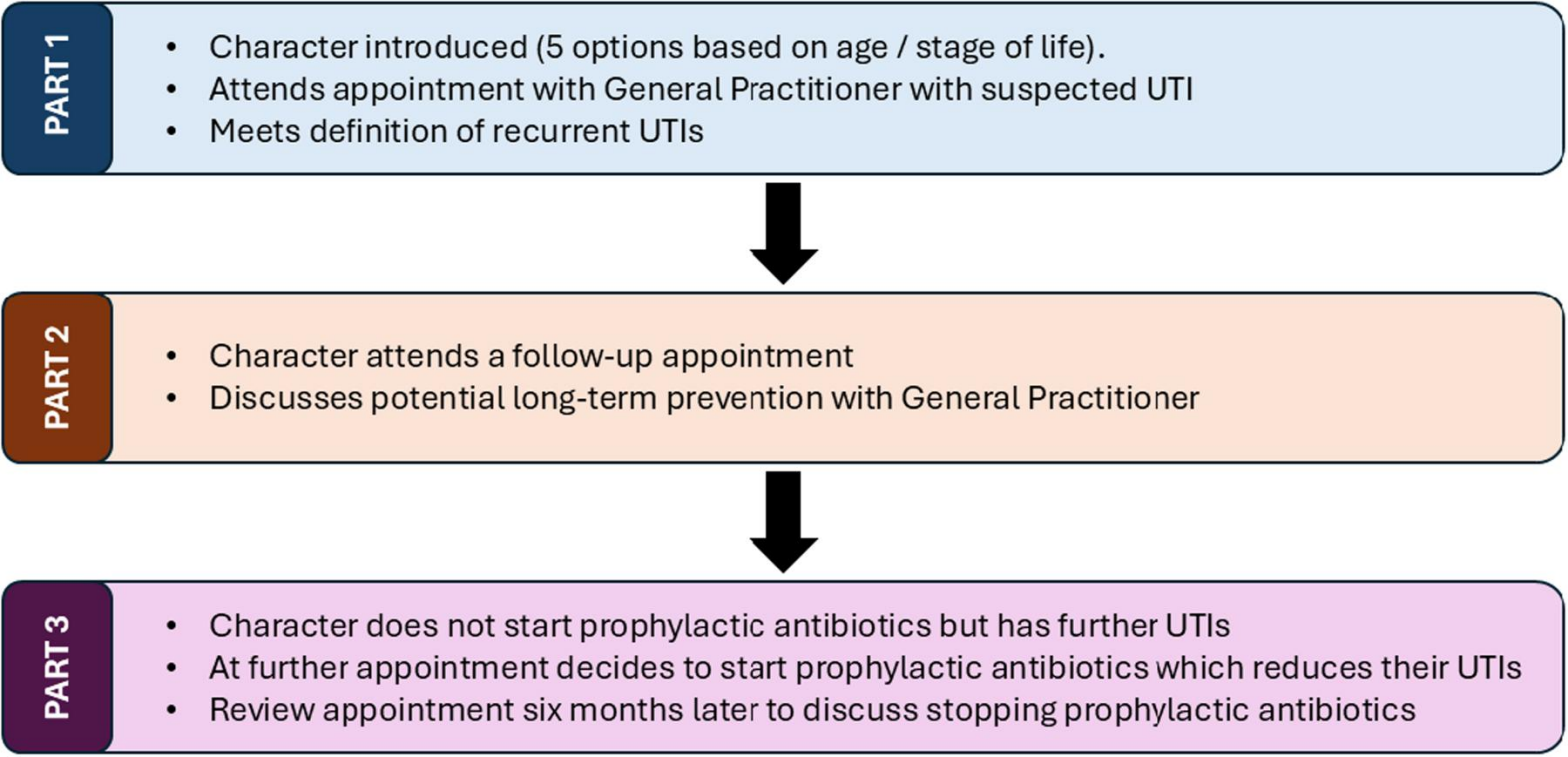
Summary of the vignette content showing the stages of the character's story.

### 2.2 Participants

Women aged 18 years and older, residing in the UK with self‐reported rUTIs (two or more UTIs in 6 months or three or more in 12 months) were eligible, excluding those with rUTI related to catheter use or pregnancy. Participants were recruited through the National Institute for Health Research (NIHR) ‘Be Part of Research’ service, which includes over 500,000 people [20]. To ensure representation from women across menopause status, participants were purposively sampled based on age (as a proxy). Menopause typically occurs between the ages of 47 and 53 years [21], so women aged below 45 years and women 45 years and older were invited separately. Those who had medically induced early menopause participated in postmenopausal groups. Participants were offered a £20 high‐street voucher for providing their time.

### 2.3 Data Collection

Participants received an information sheet and consent form to review prior to an online meeting with the lead researcher to discuss their participation and ask questions. Informed consent was provided verbally by participants, documented and was recorded on an encrypted dictaphone. Focus groups were conducted online between March and April 2023. As an ‘ice breaker’, participants described their experience of rUTIs via three words using ‘Mentimeter’ [22], which also acted as an aid to prompt discussions around the impact of their symptoms.

Focus groups were facilitated by two researchers with experience and training in qualitative research methods, who kept field notes and met regularly to discuss ideas around the data. Discussions were audio‐recorded using an encrypted digital dictaphone and transcribed verbatim by a professional transcribing company. Transcripts were reviewed for accuracy, and personal or indirect identifying information was removed. All data were stored in accordance with the Data Protection Act 2018 and General Data Protection Regulations (GDPR).

Before data collection commenced, it was anticipated that a sample of up to 32 individuals across four focus groups would be sufficient based on an assessment of the ‘information power’ [23] informed by previous UTI research [17, 24]. Our study had a focused aim, a specific sample and intended to have focus groups providing rich content. Once data collection had started, and by the fourth focus group, no new major topics or concepts were identified. However, one additional group was organised with premenopausal women, as, until this point, there were fewer participants from this sub‐group. Across the focus groups, 25 participated and seven invited participants did not attend.

### 2.4 Reflexivity

The study team included General Practitioners, a qualitative methodologist, a statistician, public health researchers, and four female public contributors with lived experience of rUTI (PPIE group). The multidisciplinary nature of the team supported consideration of professional and personal assumptions throughout the study. The study team considered the impact of the lead researcher, a male General Practitioner, conducting the focus groups. It was felt that having a clinician facilitate the discussions would be beneficial, and their role as a researcher, rather than a clinician, was discussed with participants during the consent process. Potential bias was considered by maintaining a reflexive stance throughout, with regular discussions with the second interview facilitator, a female non‐clinical researcher, after each focus group.

### 2.5 Data Analysis

Thematic analysis involved stages of generating codes and grouping into themes that reflected the concepts inherent in the data [25]. Codes were organised in QSR NVivo (March 2020). The full dataset was analysed by one researcher, and the second researcher independently coded data from two focus groups; subsequently agreeing on a thematic framework. The inductive analysis was iterative, and the final coding framework was agreed upon with the full study team. The themes were mapped to the components of the TDF for reporting. The names of some themes and subthemes were also refined during the peer review process. Quotes were identified that illustrated the meaning of the themes, ensuring representation from different individuals. Data collected from ‘Mentimeter’ were visualised into a word cloud to show examples of participants' experiences.

### 2.6 Ethical Considerations

The study was approved by the Cardiff University School of Medicine Research Ethics Committee in October 2023 (SREC reference [23]:). While the potential risk of harm was considered low for participants, there was a risk of becoming distressed when discussing rUTIs. This was minimised by using hypothetical vignettes and discussing risks during the consent process.

## 3 Results

Five focus groups were conducted with 25 women from across England, including two groups with pre‐menopausal women and three with peri‐menopausal/menopausal or post‐menopausal women. Discussions lasted 83.4 min on average (range 80–89 min). Participants provided the first part of their postcodes to identify region (Figure 2). There were participants with and without previous experience of prophylactic antibiotics (Table 1). Participants described their experience of rUTI as negative and debilitating from the ice breaker question (Figure 3). Five overarching themes with subthemes were identified from the focus groups (Table 2).

**Figure 2:**
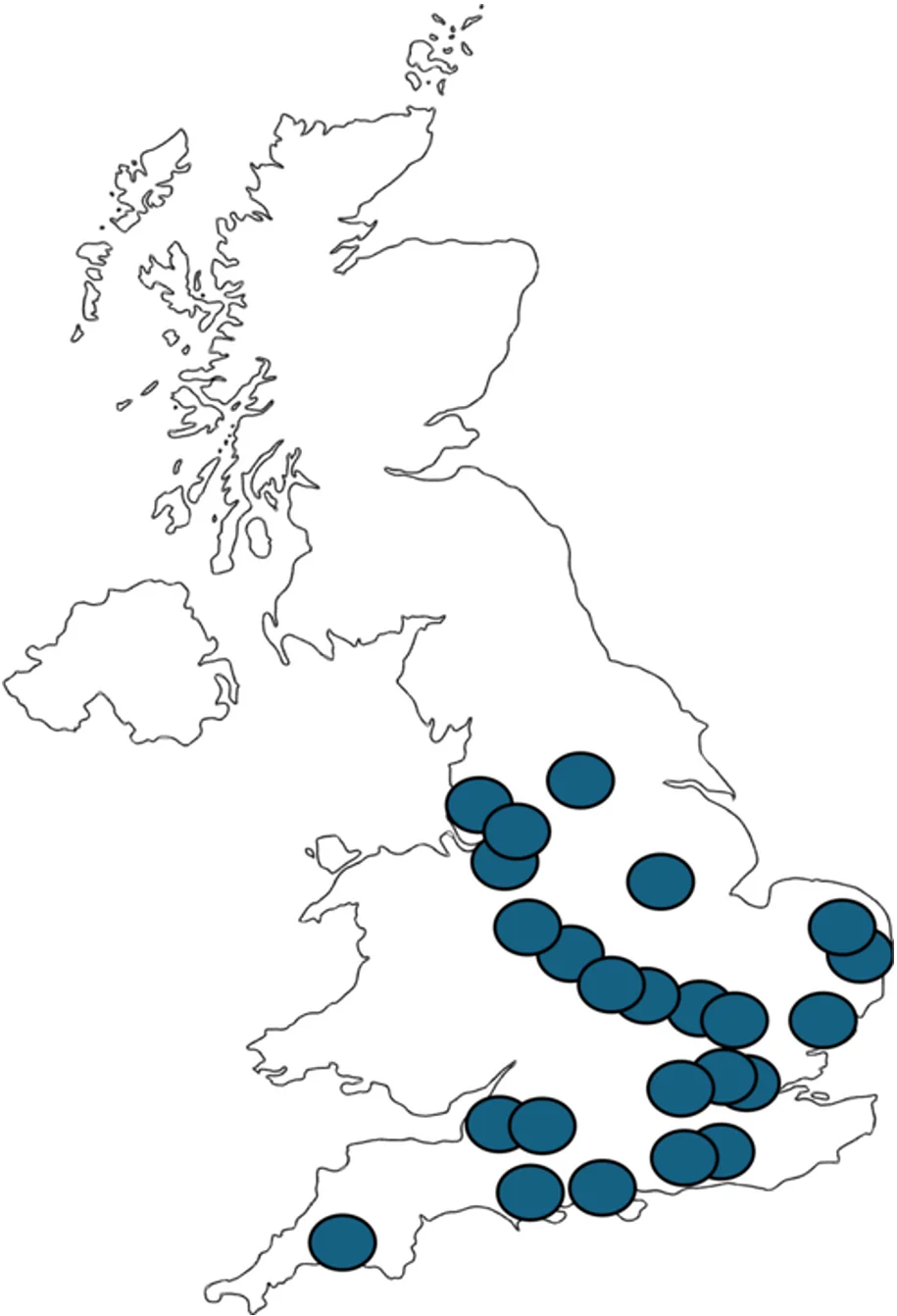
Map of the UK showing the English regions where participants lived. Please note that one region included two participants.

**Table 1:** Characteristics of participants attending focus groups (5 focus groups, n = 25).

Characteristic		*n* (%)
*Total participants*		25
Age (years)	18–27	3 (12)
	28–37	3 (12)
	38‐47	6 (24)
	48–57	2 (8)
	58–67	5 (20)
	68–77	6 (24)
Menopausal status	Pre‐menopausal	9 (36)
	Peri‐menopausal, menopausal and post‐menopausal	16 (64)
Ethnic group	White	22 (88)
	Ethnic minority group	3 (12)
Previous use of prophylactic antibiotics		16 (64)

*Note:* Specific ethnic group was provided by participants, but is grouped for reporting due to the low numbers in each group.

Abbreviation: rUTI, recurrent urinary tract infection.

**Figure 3:**
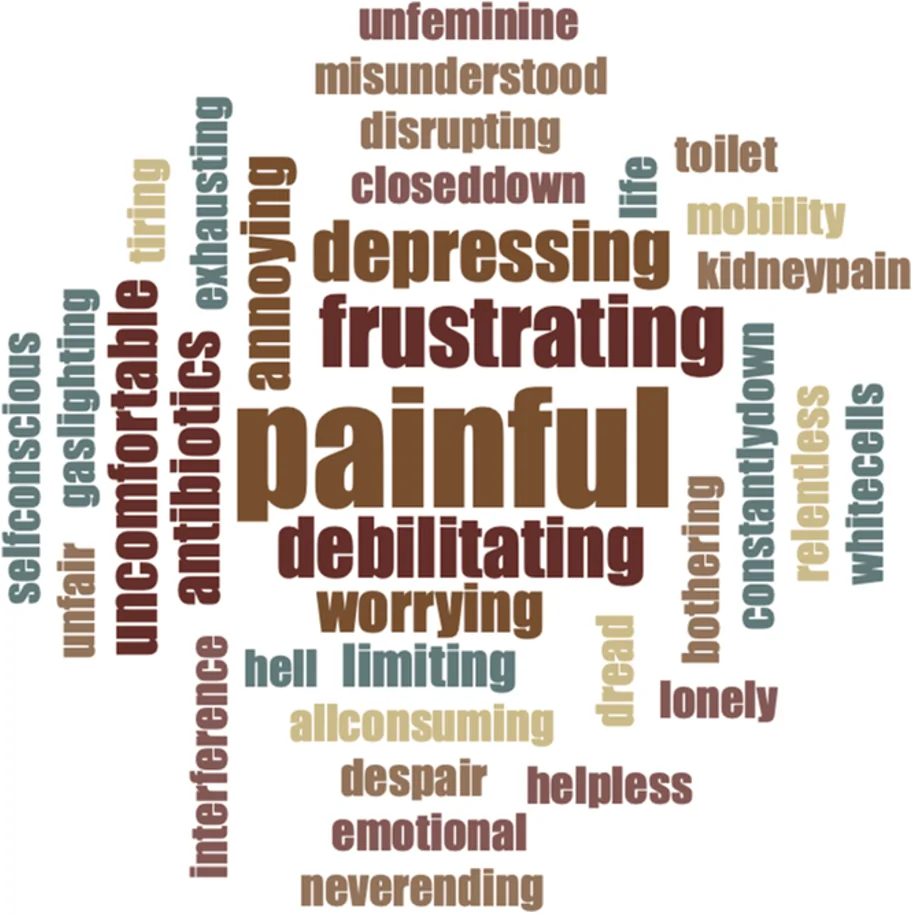
Word cloud of participants' experiences of recurrent UTIs provided by participants in the icebreaker, created using NVIVO 2020.

**Table 2:** Coding tree of the study themes and sub‐themes on the experiences of women with rUTI, linked to the Theoretical Domains Framework. rUTIs = recurrent urinary tract infections.

Themes and subthemes		Theoretical domains framework
1. Recurrent UTIs are a debilitating condition	1a. A chronic condition with symptoms that do not always fit guidelines	Social/professional role and identity Beliefs about consequences Environmental context and resources Emotion Optimism Social influences
	1b. Far reaching impact of rUTIs on daily life and the fear of further UTIs	
	1c. Information and discussion around hygiene increases stigma surrounding rUTIs	
2. Current healthcare does not support women with rUTIs	2a. Frustration and the challenge of accessing healthcare	Beliefs about capabilities Environmental context and resources Emotion Optimism Beliefs about consequences
	2b. Often feeling dismissed and unheard by healthcare professionals	
	2c. Care of rUTI should be distinct from acute episodes	
3. ‘Why is this happening?’	3a. Concern and worry about an underlying cause can be exacerbated by diagnostic tests	Beliefs about consequences Emotion Intentions Goals Environmental context and resources
	3b. Desire for further investigations and specialist referral for an underlying cause	
	3c. Perceived professional apathy about underlying cause	
4. Preventive options are frequently tried with varying success	4a. Differing views on prophylactic antibiotics for prevention and desire for the ‘right’ antibiotic	Knowledge Beliefs about consequences Emotion Goals Environmental context and resources
	4b. Concerns about side effects and antibiotic resistance	
	4c. Non‐antibiotic options are often tried, with varied success	
5. Greater support is needed for women with rUTIs	5a. Patients often feel alone and isolated, but can find support groups helpful	Social influences Emotion Memory, attention and decision‐making Environmental context and resources Social/professional role and identity
	5b. Patients want more evidence‐based information about rUTIs to support decision‐making	

### 3.1 Recurrent UTIs Are a Debilitating Condition

Women described a chronic condition, including recurring symptoms, with some experiencing persistent, long‐lasting symptoms. Most participants had a long history, often originating in childhood.

for the best part of 10 years, 300 out of 365 days for me, was in a symptomatic UTI … challenge anybody to live like that.

[FG 2, menopausal/post‐menopausal]

In most focus groups, women discussed how their symptoms didn't always fit the ‘guidelines’. Symptoms could be both physical (pain, frequency and urgency of urinating, urinating more at night) and psychological (concentration and mood changes). Some reported feeling dismissed by HCPs if they did not report specific symptoms such as pain and frequency of urination. Participants also acknowledged the difficulty for HCPs in diagnosing UTIs when there are such varying symptoms.

The doctors were very much like, are you having pain when you wee? … Is there any stinging? … my only symptom is that my back hurts, and I'm in a lot of pain, and I get a fever. And they kind of start dismissing you a bit.

[FG 1, pre‐menopausal]

Across all focus groups, women discussed the considerable impact of rUTI on their daily lives, including the impact on sleep and tiredness from passing urine at night. These, and other symptoms, had an impact on their work, social life and relationships, especially intimate relationships where rUTIs were triggered by sex. This was particularly expressed by younger women.

This condition affects everything, and it absolutely affects and devastates your personal life, especially as a younger woman.

[FG 2, menopausal/post‐menopausal]

An almost universal emotion was the fear of getting future UTIs. Women were also concerned about the timeliness of accessing medical appointments and antibiotics for future UTIs. In some cases, it was difficult to identify triggers of their symptoms, but women in most focus groups discussed the actions they would take to try and avoid them, such as avoiding sex, drinking more fluids and personal hygiene.

Recurrent UTI patients… we're some of the cleanest people on earth because we are so paranoid about getting an infection.

[FG 2, menopausal/post‐menopausal]

Participants were concerned about the potential long‐term impact of symptoms on their bodies, including the reproductive and urinary systems. Some women discussed the fear of having to live with chronic pain and that their symptoms could get worse after menopause. One group discussed the emotional impact of coming to terms with transitioning to a chronic illness:

**Participant 6**: There comes a point where you're sort of split between being relieved that somebody's taking it seriously and you're being told that this is a problem that you think you're getting recurring infections, but that's also a hard thing to hear because you've stepped into a chronic situation.

**Participant 2**: I actually struggled with that. I didn't realise I was struggling with it [laughs]. I didn't quite know what was going on, and I still am [struggling]… So it's a double‐edged thing.

[FG 3, menopausal/post‐menopausal]

Some groups discussed the stigmas associated with rUTIs, such as feeling unclean, which was often exacerbated by conflicting information found on the internet. Women in all focus groups discussed how conversations around personal hygiene with HCPs often felt judgmental, ‘patronising’, ‘demeaning’ and ‘offensive’ ‘it is like saying to a child, have you washed your hands?’ [FG 4]. Often being questioned and feeling like they had to justify toileting behaviours. This was made worse by repeated conversations of this manner each time they experienced a UTI, worsening this stigma. However, some women acknowledged it might be useful for women newer to UTIs to receive this advice.

**Participant 3**: Sometimes a doctor will say, are you sure you're wiping yourself after a stool properly front to back? Which we all do properly, because we're all conscious of that, so that's a bit patronising when they say that.

**Participant 4**: Yes, I think that's the case because I mean that's the first thing when you have a daughter that your mother tells you to do.

[FG 4, menopausal/post‐menopausal]

### 3.2 Current Healthcare Does Not Support Women With rUTIs

Women in all focus groups discussed challenges of accessing primary care appointments for UTIs, causing stress and frustration. From their regular experience with UTIs, women wanted to access treatment early, but this was often challenging, depending on when symptoms occurred. Some women discussed how they often must wait for the result of a urine sample before they can access care, and need to be ‘annoyingly persistent’ and push to be seen by a clinician. Others discussed the frustration of not being able to book a GP appointment early in the morning, resulting in limited options for alternative treatment:

**Participant 3**: If I felt fine when I woke up, but then went to the loo at 9.00 am and started to get a UTI, then I'm absolutely stumped until the next day … There is no one else to go to. You phone 111 and their advice, dependent on what symptoms, would be to go to A&E. You go to A&E, you've got a nine to 10 hour wait to see anybody…

**Participant 1**: …like [participant name] said, you try to get a doctor's appointment and you can't.

[FG 1, pre‐menopausal]

In some cases, accessing care was positive, with one participant sharing how using an online form to provide information helped save time in the consultation. Participants discussed the changing nature of general practice and how they were more likely to see a nurse or pharmacist than a GP. For most, this was a positive experience. Some also reported being referred to a community pharmacy for their UTI, though a participant suggested that this setting may be restricted in treating their UTI, as they cannot test their urine.

Participants reported mixed experiences of interacting with HCPs. Many discussed negative experiences of feeling dismissed or not listened to; one group discussed this being worse with male GPs. There were also negative experiences shared about other HCPs in both primary and secondary care. However, some participants described a positive change over time with professionals using symptoms to guide management of UTI, more than relying solely on urine tests.

Say 20 years ago, I would be categorically refused antibiotics from the GP, like some of the people have mentioned, if the tests weren't positive; however, I have seen sort of like a systemic change since then where a lot of people now are willing to treat based on symptoms.

[FG 2, menopausal/post‐menopausal]

Most participants reflected on their being a lack of continuity in their healthcare experiences, often resulting in repetitive conversations. Some experienced better continuity in secondary care, whereas others had negative experiences of being ‘passed back and forth’ between departments. They felt that women with rUTI should be viewed distinctly from acute UTIs, not ‘dealt with on a one‐off basis’ [FG 3] and should be managed as a chronic illness.

Participant 3: It can feel as if you've almost been fobbed off, you know? You went away to look at something, and then the next infection you're starting again, as if those conversations have never happened. So it is this sort of continuity, I think that somebody's working alongside you with it.

Participant 5: Yes, that's absolutely crucial, and it's so difficult to get a first appointment, and when you need a follow‐up appointment, you're back to square one.

[FG 3, menopausal/post‐menopausal]

### 3.3 ‘Why Is This Happening?’

Participants in all focus groups expressed concerns about an underlying cause for their recurrent infections, made worse by negative urine tests. Some specifically described worries about cancer. One group felt that HCPs should acknowledge when there may not be an underlying cause for the rUTI, and that even though this would not address their recurrence, it would be reassuring, so they are not constantly wondering ‘why’.

Is there something else underlying in my bladder or in my kidneys that's causing these, these continuous infections? Because you take your samples in, your samples are clear, they said, Oh no, you haven't got a UTI. And I went, well, I've got something.

[FG 1, pre‐menopausal]

All focus groups discussed specialist referrals, and most women said they would like to have further investigations. However, some women described undergoing multiple investigations, often causing more harm than good. Others, mostly pre‐menopausal women, had not been offered any investigations but wanted them. Many participants perceived there to be apathy from HCPs about UTIs and that they were a ‘problem that ladies have’ [FG 4], leading to ‘a tendency to dismiss it and not actually seek to identify the underlying causes more’ [FG 4].

If this keeps happening, what is making it happen? So is there something else going on in my body that's causing this? Is it something sinister that nobody wants to take seriously? … why does nobody think that this is a serious issue that she's [vignette protagonist] had four of them now?

[FG 1, pre‐menopausal]

### 3.4 Preventive Options Are Frequently Tried With Varying Success

Women had varying views on using prophylactic antibiotics to prevent rUTI. Some found them very effective and were therefore eager to take them despite their concerns around the risk of side effects and antibiotic resistance. In these situations, women were fearful of stopping the antibiotics, concerned their symptoms would come back and the effect this would have on their daily life. Some participants were against taking antibiotics long‐term, questioning what they were treating and concerned they may be masking an underlying cause. There were discussions about having the ‘right’ antibiotics to treat acute UTIs, which included having a longer duration. Others described being prescribed several antibiotics that did not work before landing on one that resolved the infection.

**Participant 4**: I worry often about what happens if one day I become immune to this [antibiotics], because it works absolutely brilliantly… so for me, like having these antibiotics has just been like the best thing ever …

**Participant 3**: I just find it mind‐blowing that the option given is to go on long‐term antibiotics, because who wants to take antibiotics every day if there's no reason to? And why are we even suggesting people take antibiotics every day if we don't know why it's actually happening in the first place?

[FG 1, pre‐menopausal]

Across focus groups, women had either experienced or were concerned about side effects of antibiotics or developing antibiotic‐resistant infections. Specific side effects experienced by participants included gastrointestinal symptoms, thrush, skin reactions and antibiotic resistance. Some participants reported taking probiotics or making dietary changes to address side effects.

When I was on Nitrofurantoin… that was the only one that I could take without horrendous constant nausea and side effects, and also thrush.

[FG 1, pre‐menopausal]

Women in all focus groups discussed alternatives to antibiotics to prevent rUTIs; these included methenamine hippurate, vaginal oestrogen, cranberry, d‐mannose and Chinese herbal medicine. For some, they effectively reduced their recurrence, or they knew that it helped other women; others found no effect. Some women were interested in taking them where the standard treatment had been ineffective, while others were more dismissive of their usefulness. Some women said they would try alternatives before taking prophylactic antibiotics if there were fewer side effects. Women believed they might take time to be effective and that they would consider using them alongside antibiotics in the first instance, and then continue their use after stopping antibiotics.

I guess I have like a massive success story…I started taking the Hiprex [methenamine hippurate] and I haven't had an infection since, and I occasionally get very like slight symptoms that don't result in anything. I consider that like an absolute win.

[FG 5, pre‐menopausal]

### 3.5 Greater Support Is Needed for Women With rUTIs

Most participants described the need for peer support and feeling isolated with rUTIs. While some discussed seeking support from friends, family or work colleagues, many did not feel able to as they felt there was little they could do to help, they didn't want to burden them, or a perception that UTIs were not openly discussed. Participants felt that it was not generally known how common rUTIs are and that providing information on this could help them feel less alone. Support groups were viewed as useful to share experiences and recommendations for prevention, but there were some concerns around the reliability of the information shared.

It's not something that people talk about. They say they've got the flu or they've got migraine or something… you don't talk about the bladder, because it's a very personal thing (laughs) isn't it, and people really can't relate to it.

[FG 3, menopausal/post‐menopausal]

Participants reported accessing information online, often because not enough was provided by HCPs, but again were concerned about the accuracy. They wanted an active involvement in decisions around prevention, but to be given enough evidence‐based information around effectiveness and risks to make this decision, including ways to identify their personal priorities and how these might be affected by treatments. Receiving this information before consultations would be useful due to time constraints in the consultation.

The GP was like, you could take a low course, longer course, you could do this, it could be that…I needed and would have wanted some assurance, or like some clearer direction about what I should do…although it's great to have a voice in your own care, it's like I don't know what to do, I'm not an expert now…I'm really looking for some guidance from the GP.

[FG 1, pre‐menopausal]

I'd want to know kind of numbers, I'm very data driven, I'd want to know numbers and, and stats of how many people this helps, how many people get side effects from this, versus this … so that I can compare.

[FG 5, pre‐menopausal]

Participants felt there was a need for better support when the decision is made to stop prophylactic antibiotics. They felt it would be useful to have a plan of what to do if the UTIs came back, for example, a follow‐up appointment and an ‘emergency pack’ of antibiotics, so they did not experience delays in accessing healthcare. They also felt it was important to be forewarned about the possibility of stopping prophylactic antibiotics, as the review appointment could often feel like a ‘high‐pressure situation’. Similarly, participants felt that discussions around long‐term prevention should not occur at the same time as experiencing an acute UTI, as their main goal was to treat the acute infection.

When you go to the GP, and you've got a full‐on UTI going on, you don't always think that clearly, you just want the antibiotics and you want to get out, you don't want to have long conversations.

[FG 2, menopausal/post‐menopausal]

### 3.6 Themes in the Context of the Theoretical Domains Framework

When interpreting themes using the TDF, it was apparent that behaviours were influenced by interactions between several domains across cognitive, social, emotional and contextual factors. Emotional responses cut across all themes, each influencing the goal of participants (to prevent future recurrences) and in decisions around prevention, such as fear of stopping antibiotic prophylaxis when it had proved effective. While beliefs about consequences influenced concerns about the use of antibiotics, decisions around prevention were juxtaposed between the threat of AMR and the fear of recurrences. Contextual factors, such as the difficulty of getting an appointment, also influenced this, as women did not want to be back in a position where they would struggle to access prompt treatment:

Probably some dread…of what's about to happen in the next few days. Is she [vignette protagonist] going to be able to get the antibiotics or a GP appointment in time again? How long are they going to take to work? How many days of the antibiotics are there?.

[FG 1, pre‐menopausal]

Nevertheless, women were willing to try non‐antibiotic options if they had proven effectiveness or could be used alongside antibiotics or after stopping prophylaxis. Social and professional identity affected women's experiences with rUTI management, sometimes negatively impacting the decision‐making process with HCPs, as they often could not get their voice heard or received inadequate advice on prevention. An example of the interplay between knowledge, social and professional identity and environmental barriers is where a participant discussed how HCPs did not make them aware of the link between rUTIs and the menopause:

It [menopause] was never talked of to me until I researched myself the effect on the urinary tract, of the menopause.

[FG 4, menopausal/post‐menopausal]

Other environmental barriers included the lack of continuity in the healthcare environment and the lack of useful information they can trust, making decisions around preventive options difficult, leading to women experimenting with different options with little guidance. An example of the impact of professionals on women's emotions, as well as the context of rUTI being treated without continuity, was around the impact of repeated advice on hygiene:

I think with that conversation it's something that kind of happens every time that I went to the doctor about my UTIs … there's a point where it feels patronising even if it isn't intended in that way and like the way I have to go to the doctor and justify which way like I wipe after I go to the toilet … it's kind of demeaning.

[FG 5, pre‐menopausal]

## 4 Discussion

### 4.1 Summary

This qualitative study identified that women experience rUTIs as a chronic condition and require greater continuity and proactive management, with care delivered differently from that of acute episodes. Women often struggled to access care and described healthcare interactions as negative, experiencing repetitive discussions around hygiene that exacerbate stigma around UTIs. Women were concerned about an underlying cause and wanted more specialist referrals and engagement from HCPs on further investigations. Views and experiences of antibiotic and non‐antibiotic preventive strategies were mixed, but there was significant concern around stopping prophylactic antibiotics if it meant that symptoms could return. Experiencing rUTIs was isolating, and women require greater support in discussions about prevention, particularly evidence on risks and benefits of different preventive treatments to aid decision‐making.

### 4.2 Strengths and Limitations

The recruitment achieved good representation across age and menopause status of women; as rUTIs tend to be more prevalent in women post‐menopause, there is potential that pre‐menopausal women could be underrepresented. There were also participants with and without experience of prophylactic antibiotics, which gave a balanced discussion on their use. Vignettes were used effectively to support participants with a subject matter that could be distressing, and the involvement of the PPIE group in their development ensured relevance.

A limitation of recruitment is that information on socioeconomic status and health literacy levels was not collected or used to purposively sample participants. There may be selection biases through using the ‘Be Part of Research’ panel, as patients who have signed up to this panel may have certain characteristics which differ from those not on the panel, and those without internet access. Participants were all from England despite the panel spanning the UK, and it is possible that those from different levels of deprivation may not have been represented. While online focus groups offered practical advantages, evidence suggests they may yield less in‐depth data and weaker participant interaction than face‐to‐face groups [26]. Although sampling aimed to recruit participants from diverse ethnic groups, only three minoritised individuals participated. Further work should engage participants from diverse backgrounds and those experiencing deprivation who may have different needs around prevention for rUTIs. The focus groups were conducted in the English language; further studies could engage with specific communities that may need tailored information or support, for example, non‐English speaking.

### 4.3 Comparison to Existing Literature

Women in our study described their symptoms not fitting ‘guidelines’ and that this often led to them feeling dismissed by clinicians. Previous research identified that women with rUTIs often experience atypical symptoms [27, 28]. The severe impact on quality of life aligns with previous research [6], with our findings building on this with a strong consensus that the condition should be managed distinctly from those with acute UTIs. This aligns with another study where women discussed inconsistent management and the impact that different clinicians have on conflicting care and advice [29].

Stigma surrounding UTI has been identified across international studies, linked to internal shame, and its associations with female health, sexual activity, and cleanliness [14, 30, 31, 32, 33]. Other studies describe how stigma can be a barrier to seeking healthcare and negatively impact interactions with clinicians [14]. Our findings align with these, with the novel finding of the impact of repeated hygiene advice, which was perceived as demeaning and exacerbated stigma associated with cleanliness. Considering the lack of evidence on the link between hygiene and rUTI [10], clinicians could approach this advice with sensitivity, and it may be more beneficial for women experiencing UTI for the first time.

Many women in our focus groups described feeling dismissed or not listened to in healthcare interactions. This is a common theme in the literature for women with urinary symptoms who often feel their symptoms are trivialised [31, 33]. Linked to this, women often prefer engaging with female clinicians [15]. Since writing up our findings, a qualitative study has been published which explored patients' experiences navigating UK healthcare for rUTI [12]. Our findings align with rUTI management characterised by patients needing to advocate for themselves. Our findings complement and extend the study by explicitly focusing on women's experiences of preventive management and navigating decisions with HCPs, offering recommendations for decision support needs. This study, like others, has also highlighted how patients struggle with being referred or investigated for underlying causes [6, 12, 14]. As identified in our discussions, this exacerbated women's feelings of being dismissed as they felt clinicians did not take their condition seriously. Patient concerns around the unexplained cause of recurrences, such as bladder diseases or cancer, are a common theme in other literature [29].

The complex feelings around antibiotics that women described have been noted in other research, including concerns over their use and the revolutionary effect it can have on their quality of life [6, 31]. A novel area of our discussions concerned the process of stopping antibiotics. Healthcare professionals have reported that patients may resist the deprescribing of antibiotics [34] and often fear or question the appropriateness of stopping medications [35]. Conversely, some women in our focus groups questioned why antibiotics were being used and whether they may be masking something more sinister.

Almost all women in our focus groups discussed trying non‐antibiotic prevention strategies, with mixed success [15]. Some found success with strategies like d‐Mannose, cranberry or probiotics, which have anecdotal effectiveness, but some conflicting evidence [36, 37, 38]. While not explicitly recommended by NICE in the UK, they do acknowledge patients may wish to try them, and other international guidelines acknowledge cranberries as an option [39]. Women tended to have positive experiences with methenamine and vaginal oestrogen, which have stronger evidence of effectiveness. Older women described how the link between rUTI and the menopause was often not discussed by clinicians. Overall, women across focus groups discussed wanting more information about preventive options. Pacho and colleagues discussed the need for self‐care not to be separate from professional healthcare, ensuring the role of clinicians is clear in advising patients. This is particularly important for women from racialised minorities and low‐income households who face inequalities in accessing healthcare and potential barriers to practising self‐care [15]. The recent study by Newlands and colleagues also highlighted that patients wish to have more information on non‐antibiotic options, and the authors recommended decision aids to support discussions on prevention [12]. Existing patient information leaflets and decision aids for uncomplicated UTI support conversations between clinicians and patients on acute treatment options, self‐care and safety netting [17, 24].

### 4.4 Recommendations and further Research

Table 3 summarises recommendations based on the themes of this study. The use of the TDF highlighted that women's decisions were not solely knowledge‐based but also represented intersecting factors across key domains like emotion and context. Barriers across the environmental context and social and professional identity also demonstrated that behaviours are constrained by healthcare system barriers, and not just individual choices. Therefore, systematic changes to management need to be considered so that patients with rUTIs are treated as a separate group and condition from people presenting with acute episodes. Emotional responses were one of the most influential TDF domains, cutting across all themes, with participants expressing anxiety and fear of further recurrences and plagued by the question ‘why is this happening’, guiding their goals for referrals to investigate an underlying cause. Consequently, clinicians should explain the rationale for (or not) arranging further investigation and referrals for rUTI patients. As highlighted by the TDF analysis, there is a conflict between concerns about antibiotic resistance and patients' inherent goals to reduce recurrences and fear around stopping antibiotics when they have proven effective. Clinicians could support them through clarity on the expected length of time for prophylaxis, providing ‘rescue’ packs and reassurance about accessing care if there is a recurrence upon cessation.

**Table 3:** Recommendations for how discussions about rUTI prevention could be better supported in practice, mapped to themes and the TDF.

Themes	Relevant TDF domain(s)	Recommendations for practice
Recurrent UTIs are a debilitating condition	– Emotion – Beliefs about consequences	– Careful consideration of messaging around hygiene for patients with rUTIs, especially when evidence is limited.
Current healthcare does not support women with rUTIs	– Environmental context and resources – Social influences – Social/professional role and identity	– Clear identification and coding of patients with rUTIs to guide assessment and preventive management discussions outside of acute episodes and prevent referral to inappropriate services.
	– Environmental context and resources – Social influences – Social/professional role and identity	– Proactive discussions around rUTI management, which should not only occur when patients are actively experiencing acute episodes. – Key stages for discussion are when patients move from acute episodes to a recurrent presentation, when discussing the risks and benefits of preventive options and when reviewing or changing prevention treatment (e.g. antibiotics).
‘Why is this happening?’	– Knowledge – Belief about consequences	– Provision of information about red flags (for serious underlying cause, including cancer) and when investigation is advised.
	– Emotion – Knowledge	– Clarity on when referral for investigation is, and is not, recommended.
Preventive options are tried with varying success	– Memory attention and decision‐making – Knowledge	– Clear guidance on self‐help options and levels of evidence.
	– Knowledge – Beliefs about capabilities	– Ask patients about the preventive options they have already tried, as many patients are likely very knowledgeable and confident with various self‐care measures.
	– Beliefs about consequences	– Provision of clear, balanced evidence of the increased risks of prophylactic antibiotics.
	– Environmental context and resources – Goals	– Consideration of a ‘rescue pack’ of antibiotics when supporting a trial off prophylactic antibiotics.
Greater support is needed for women with rUTIs	– Memory, attention and decision‐making	– Development of decision‐making support tools for rUTIs.
	– Social influences – Emotion	– Increased awareness of the prevalence of rUTIs to normalise experiences and reduce isolation.
	– Social influences – Memory attention and decision‐making – Environmental context and resources	– Directing patients to reliable online information and support groups.

A patient decision aid for rUTI prevention could address several barriers by supporting patients with evidence‐based information to facilitate shared decision‐making. However, many of the challenges and barriers identified in this study would not be addressed by a decision aid alone and would require additional interventions and further research. A patient diary may be another potentially useful intervention to track symptoms, identify triggers and to monitor the effectiveness of self‐care strategies, as patients highlighted barriers with this. Further work is required to ensure clear coding for rUTI in patients' records to help prevent them from being directed to inappropriate services. For example, community pharmacies in England can supply medications, including antibiotics, for some patients with acute UTIs, and rUTI patients would not currently meet the inclusion criteria for this service. Exploring additional prompts on GP systems may help to flag where patients require a review of their treatment, for example, if they have evidence of resistant infections. Clinicians could consider that patients with rUTI may present differently from patients with acute infections, but the reasons for this are unclear. Further research is needed in this area to ensure current UTI prescribing guidelines are also appropriate for this population.

## 5 Conclusions

There are several opportunities to improve the prevention and management of rUTIs in primary care and improve the experiences of patients. Patients with this condition require greater continuity, proactive discussions on prevention and should be managed differently from those with acute episodes. To support effective decision‐making around prevention, complex interventions and strategies are required to address the environmental, social, and emotional context of rUTI. A patient decision aid represents one way to support discussions on the risks and benefits of different preventive options and enhance shared decision‐making.

## Author Contributions

**Catherine V. Hayes:** investigation, formal analysis, methodology, validation, visualisation, writing – original draft, writing – review and editing. **Donna M. Lecky:** formal analysis, methodology, writing – review and editing. **Haroon Ahmed:** conceptualisation, formal analysis, methodology, project administration, writing – review and editing. **Rebecca Cannings‐John:** conceptualisation, formal analysis, methodology, project administration, writing – review and editing. **Adrian Edwards:** conceptualisation, writing – review and editing, methodology, formal analysis, project administration. **Fiona Wood:** conceptualisation, writing – review and editing, methodology, formal analysis, project administration. **Leigh Sanyaolu:** conceptualisation, data curation, formal analysis, funding acquisition, investigation, methodology, project administration, visualisation, writing – original draft, writing – review and editing.

## Ethics Statement

The study was approved by the Cardiff University School of Medicine Research Ethics Committee (SREC reference [23]/66).

## Consent

Informed consent was obtained remotely from all participants for participation in this qualitative study and its publication; the consent process was documented in written form and audio‑recorded.

## Conflicts of Interest

The authors declare no conflicts of interest.

## Supporting information


Supporting File 1

Supporting File 2

## Data Availability

The data that support the findings of this study are not publicly available. Specifically, participants did not provide consent for their data to be shared beyond the research team.
