# Supplementary material for: Women's Experiences of Recurrent UTIs: A Qualitative Study of Preventive Management and Decision Support Needs
Source: Health Expect. 2026 Oct 3;29(5):e70890. doi: 10.1111/hex.70890 (PMC13633698; doi:10.1111/hex.70890)
Supplement: Supplementary file 1 [file HEX-29-e70890-s001.docx]

# Supplementary material 2

## COREQ (COnsolidated criteria for REporting Qualitative research) Checklist

| **Topic** | **Item No.** | **Guide Questions/Description** | **Reported on**  **Line No.** |  |  |  |
| --- | --- | --- | --- | --- | --- | --- |
| **Domain 1: Research team**  **and reﬂexivity** | | | |  |  |  |
| *Personal characteristics* | | | |  |  |  |
| Interviewer/facilitator | 1 | Which author/s conducted the interview or focus group? | [researcher 1 – corresponding author] conducted the interviews, with [researcher 2 – first author] supporting.  Reported in methods, data collection. |  |  |  |
| Credentials | 2 | What were the researcher’s credentials? E.g. PhD, MD | [researcher 1] has a post-graduate diploma in Epidemiology.  [researcher 2] has a master’s degree in public health (MPH) |  |  |  |
| Occupation | 3 | What was their occupation at the time of the study? | [researcher 1] was a General Practitioner and Doctoral Fellow at the time of the interviews.  [researcher 2] is a public health researcher.  Reported in methods, reflexivity |  |  |  |
| Gender | 4 | Was the researcher male or female? | [researcher 1] is male  [researcher 2] is female  Reported in methods, reflexivity |  |  |  |
| Experience and training | 5 | What experience or training did the researcher have? | Both researchers have undertaken training in qualitative research methods.  Reported in methods, data collection. |  |  |  |
| *Relationship with*  *Participants* | | | |  |  |  |
| Relationship established | 6 | Was a relationship established prior to study commencement? | Researchers had no prior relationship with any participant. |  |  |  |
| Participant knowledge of  the interviewer | 7 | What did the participants know about the researcher? e.g. personal  goals, reasons for doing the research | Participants knew the professional role of researchers and the purpose of the research |  |  |  |
| Interviewer characteristics | 8 | What characteristics were reported about the inter viewer/facilitator?  e.g. Bias, assumptions, reasons and interests in the research topic | [researcher 1] has an interest in recurrent UTIs and this research was to inform future work, including the potential development of a patient decision aid. |  |  |  |
| **Domain 2: Study design** | | | |  |  |  |
| *Theoretical framework* | | | |  |  |  |
| Methodological orientation and Theory | 9 | What methodological orientation was stated to underpin the study? e.g. grounded theory, discourse analysis, ethnography, phenomenology, content analysis | No underlying theory was used as this study was an exploratory piece of work. The TDF was used in the development of the topic guide and interpretation of themes.  Reported in methods, study design. |  |  |  |
| *Participant selection* | | | |  |  |  |
| Sampling | 10 | How were participants selected? e.g. purposive, convenience,  consecutive, snowball | A purposive sample of women with recurrent UTIs was recruited to ensure representation based on menopausal status.  Reported in methods, participants. |  |  |  |
| Method of approach | 11 | How were participants approached? e.g. face-to-face, telephone, mail,  email | Participants were approached via the NIHR Be Part of Research UK-wide registry. Potential participants were sent information about the study via email.  Reported in methods, participants. |  |  |  |
|  |  |  |  |  |  |  |
| Sample size | 12 | How many participants were in the study? | 25 women with self-reported recurrent UTIs participated in the study, as reported in results. |  |  |  |
| Non-participation | 13 | How many people refused to participate or dropped out? Reasons? | 7 participated did not attend scheduled focus groups.  Reported in methods, data collection. |  |  |  |
| *Setting* | | | |  |  |  |
| Setting of data collection | 14 | Where was the data collected? e.g. home, clinic, workplace | Focus groups took place online using Microsoft Teams.  Reported in methods, data collection. |  |  |  |
| Presence of non-  participants | 15 | Was anyone else present besides the participants and researchers? | Only researcher 1 and 2 and the participants were present during interviews. |  |  |  |
| Description of sample | 16 | What are the important characteristics of the sample? e.g. demographic data | Age, ethnic group, UK region, menopausal status and history of antibiotic prophylaxis were collected from participants and summarised in results, Table 1. |  |  |  |
| *Data collection* | | | |  |  |  |
| Interview guide | 17 | Were questions, prompts, guides provided by the authors? Was it pilot tested? | A topic guide with prompts was developed alongside five vignettes with hypothetical scenarios features characters of different ages and menopausal status. The vignette selected for each focus groups was based on the group characteristics. The topic guide and vignettes were developed with input from supervisors, public contributors and collaborators.  Reported in methods, study design. |  |  |  |
| Repeat interviews | 18 | Were repeat interviews carried out? If yes, how many? | N/A – no repeat interviews required. |  |  |  |
| Audio/visual recording | 19 | Did the research use audio or visual recording to collect the data? | Focus groups were audio-recorded. |  |  |  |
| Field notes | 20 | Were ﬁeld notes made during and/or after the interview or focus group? | Yes, both during and after focus groups. |  |  |  |
| Duration | 21 | What was the duration of the interviews or focus group? | The average duration of focus group interviews was 83.4 minutes (range 80-89 minutes). |  |  |  |
| Data saturation | 22 | Was data saturation discussed? | No new themes or concepts, related to the study objectives, were identified after focus group four.  Reported in methods, data collection. |  |  |  |
| Transcripts returned | 23 | Were transcripts returned to participants for comment and/or correction | N/A- transcripts were not returned to participants. |  |  |  |
| **Domain 3: analysis and**  **ﬁndings** | | | |  |  |  |
| *Data analysis* | | | |  |  |  |
| Number of data coders | 24 | How many data coders coded the data? | One researcher coded all data, a second researcher coded 25% (double coded) |  |  |  |
| Description of the coding  tree | 25 | Did authors provide a description of the coding tree? | Yes, table 2 |  |  |  |
| Derivation of themes | 26 | Were themes identiﬁed in advance or derived from the data? | Study themes were derived from the data. |  |  |  |
| Software | 27 | What software, if applicable, was used to manage the data? | NVivo, March 2020. |  |  |  |
| Participant checking | 28 | Did participants provide feedback on the ﬁndings? | No. |  |  |  |
| *Reporting* | | | |  |  |  |
| Quotations presented | 29 | Were participant quotations presented to illustrate the themes/ﬁndings?  Was each quotation identiﬁed? e.g. participant number | Illustrated in results section with details of the focus group and their menopausal status against quotations. |  |  |  |
| Data and ﬁndings consistent | 30 | Was there consistency between the data presented and the ﬁndings? | Illustrated in results and discussion sections |  |  |  |
| Clarity of major themes | 31 | Were major themes clearly presented in the ﬁndings? | Illustrated in results section |  |  |  |
| Clarity of minor themes | 32 | Is there a description of diverse cases or discussion of minor themes? | Illustrated in results section |  |  |  |

Developed from: Tong A, Sainsbury P, Craig J. Consolidated criteria for reporting qualitative research (COREQ): a 32-item checklist for interviews and focus groups. *International Journal for Quality in Health Care*. 2007. Volume 19, Number 6: pp. 349 – 357
