# Supplementary material for: Women's Experiences of Recurrent UTIs: A Qualitative Study of Preventive Management and Decision Support Needs
Source: Health Expect. 2026 Oct 3;29(5):e70890. doi: 10.1111/hex.70890 (PMC13633698; doi:10.1111/hex.70890)
Supplement: Supplementary file 2 [file HEX-29-e70890-s002.docx]

**Focus group schedule for women with recurrent UTIs and clinical vignettes.**

# Focus group schedule.

**Introduction**

Good morning/afternoon and welcome to this focus group on recurrent urine infections. Thank you for agreeing to participate and give up your time to help us with this study. I am [researcher 1 name], I am a GP and I am doing a PhD to try and improve discussions between women and their healthcare professionals on the management of recurrent UTIs. This is my colleague [researcher 2 name], and I will let her introduce herself.

**Aims of focus group**

This focus group is to get your valuable views and opinions on what information women with recurrent urine infections would like when discussing treatments to prevent their recurrent urine infections and how we could improve these discussions.

**Session format**

During this focus group we will discuss an imaginary scenario of a woman with recurrent urine infections that has gone to see her GP. The scenario is in three parts and after each part I will stop to get your thoughts and views on what the woman in the scenario may be thinking. You can also give your own experiences if you would like to. After the scenario I would like to discuss the decision aids that I have sent to you in advance to see what you think of the layout, information etc. I will be recording our discussions during this focus group on a Dictaphone as highlighted in the information sheet. Anything discussed in this focus group will be kept confidential and anything you say will not be able to be traced back to you.

Does anyone have any questions about this before we start?

**Ground rules**

It is also important we discuss some ground rules for this focus group. We will be potentially discussing some sensitive and personal experiences and therefore it is important that anything discussed is not discussed outside this focus group and is kept confidential. Again, this is in the information sheet, and we discussed this when you consented to be involved in this study. Please also be respectful about other people’s views even if they differ to your own and try to not interrupt or talk over each other during the discussions.

Does anyone have any questions?

I would now like to ask you all in turn to introduce yourself and give a sentence why you were interested to be involved in this study.

Thank you everyone for introducing yourselves.

**Ice breakers**

If there are no further questions, I will now start the recorder [**Dictaphone recording starts**].

Before we get started with the scenario, I would like you to answer a question for me please. If you go to menti.com and type in the following code [XXXXX], then answer the following question – “in three words, can you describe your experience of recurrent UTIs”.

Excellent, thank you. I will come back to these points during our discussions.

For the scenario we are discussing today, we need a name for our character, does anyone have any suggestions?

Thank you, so we will now discuss [name suggested]’s scenario.

**[one of the clinical vignettes will be selected below depending on the participants in the focus group. Potential questions and prompts are included below each part to address the study objectives if this information is not discussed by the focus group].**

**[discuss clinical vignette]**

Thank you so much for sharing your thoughts and views around [name]’s story. This will be very valuable for our study. Now I would like to discuss the three decision aids which I have sent to you in advance. I am most interested in finding out your views on the design, the structure and NOT the condition it relates to.

Specific areas to ask the focus group to discuss if haven’t:

- The structure of the decision aid:
  - How is the information presented?
  - How are the benefits and risks of treatment options presented?
- The format of the decision aid i.e., paper/ electronic/ web page
- Preference for the design or structure of a decision aid 1 or 2 or the decision support tool?
- Views on the sections about deciding what is important to you and making the decision.
- The positives and negatives of decision aid 1, then decision aid 2 and finally the decision support tool.
- Preference for one decision aid over the other, why?

# Clinical scenarios

## 1. 20-year-old – pre-menopausal.

**[name]** is 20 years old and is currently at university studying history. She is not currently in a relationship and lives with two friends in a student house. Over the last year **[name]** has had three urine infections and three days ago she started noticing it was painful to pass urine and was passing urine more frequently. She thinks she has another urine infection and tries drinking more fluids, but this does not help. **[name]** sees Dr Morgan (GP) who agrees she has a urine infection and prescribes antibiotics. This is the fourth infection **[name]** has had in the last 12 months and she is worried about them. **[name]** thinks they are more likely to occur when she is stressed. They discuss hygiene and water intake as ways to prevent infections and a follow-up appointment made.

**What do you think is going through [name]’s mind? [TDF 1 Knowledge]**

**Is there anything [name] could be worried about? [TDF 1 Knowledge, 13 Emotion]**

**What do you think [name] will want to ask the GP at the follow-up appointment? [TDF 6 Beliefs about Consequences]**

**What information do you think [name] wants about recurrent urine infections? [TDF 1 Knowledge]**

**How do you think [name] might want to get this information? [TDF 11 Environmental Context and Resources]**

**[name]** has had a further infection prior to her follow-up appointment despite doing what they discussed at the last appointment. Dr Morgan discusses one of the potential treatments that could reduce the number of infections is taking low dose long-term antibiotics once a day at night. **[name]** is concerned about taking daily antibiotics as she has heard about the risk of antibiotics not working in the future. She asks Dr Morgan about any antibiotic alternative treatments. Dr Morgan says that there are a few other treatments but there is differing information on how good they are at preventing further infections.

**What do you think is going through [name]’s mind now? [TDF 1 Knowledge]**

**What information do you think [name] will want to know about taking antibiotics to prevent urine infections when making this decision? [ TDF 1 Knowledge, 9 Goals, 10 Memory, Attention and Decision Processes]**

**What information [name] will they want on these non-antibiotic treatments when making this decision? [ TDF 1 Knowledge, 9 Goals, 10 Memory, Attention and Decision Processes]**

**How might she want the information to help her make this decision? [TDF 13 Emotion]**

**Is there anything you think [name] might be concerned or worried about? [TDF 13 Emotion]**

**[name]** decides not to start long-term antibiotics and. Six months later **[name]** makes another appointment and sees Dr Hussein as she has had another three urine infections despite using a variety of over-the-counter treatments. They discuss her infections and **[name]** is keen to start long-term antibiotics as her infections are really impacting her studies. The antibiotics work well and **[name]** has less urine infections over the next six months and has another appointment with Dr Morgan to review her long-term antibiotics and whether to continue or stop them.

**What do you think [name] is thinking prior to attending this appointment? [TDF 1 Knowledge, 6 Beliefs about Consequences]**

**What information do you think [name] will need to make this decision with her GP? [TDF 1 Knowledge, 9 Goals, 10 Memory, Attention and Decision Processes]**

**How and when would it be best for [name] to receive this information? [TDF 11 Environmental Context and Resources]**

**How could [name] be supported by her GP to make this decision? [TDF 3 Social/Professional Role and Identity, 6 Beliefs about Consequences, 11 Environmental Context and Resources, 12 Social Influences]**

**Do you think [name] would want different information at this review compared to when she was deciding whether to start long-term antibiotics? [TDF 1 Knowledge, 11 Environmental Context and Resources]**

## 2. 37-year-old – pre-menopausal

**[name]** is 37 years old. She is married and has two young children aged 2 and 6. Over the last year **[name]** has had three urine infections and three days ago she started noticing it was painful to pass urine, was passing urine more frequently and noticed her urine had started to smell. She thinks she has another urine infection and tries drinking more fluids, but this does not help. **[name]** sees Dr Morgan (GP) who agrees she has a urine infection and prescribes antibiotics. This is the fourth infection **[name]** has had in the last 12 months and she is worried about them. **[name]** thinks they are more likely to occur after sexual intercourse. They discuss hygiene and water intake as ways to prevent infections and a follow-up appointment made.

**What do you think is going through [name]’s mind? [TDF 1 Knowledge]**

**Is there anything [name]could be worried about? [TDF 1 Knowledge, 13 Emotion]**

**What do you think [name] will want to ask the GP at the follow-up appointment? [TDF 6** **Beliefs about Consequences]**

**What information do you think [name] wants about recurrent urine infections? [TDF 1** **Knowledge]**

**How do you think [name] might want to get this information? [TDF 11** **Environmental Context and Resources]**

**[name]** has had a further infection prior to her follow-up appointment despite doing what they discussed at the last appointment. Dr Morgan discusses potential treatments that could reduce the number of infections including taking an antibiotic after intercourse or taking low dose long-term antibiotics once a day. **[name]** is concerned about taking long-term antibiotics as she has heard about the risk of antibiotics not working in the future. **[name]** asks Dr Morgan about any antibiotic alternative treatments. Dr Morgan says that there are a few other treatments but there is differing information on how good they are at preventing further infections.

**What do you think is going through [name] mind now? [TDF 1** **Knowledge]**

**What information do you think [name] will want to know about taking antibiotics to prevent urine infections when making this decision? [ TDF 1** **Knowledge, 9** **Goals, 10** **Memory, Attention and Decision Processes]**

**What information [name] will they want on these non-antibiotic treatments when making this decision? [ TDF 1** **Knowledge, 9** **Goals, 10** **Memory, Attention and Decision Processes]**

**How might she want the information? [TDF 13** **Emotion]**

**Is there anything you think [name] might be concerned or worried about? [TDF 13** **Emotion]**

**[name]** decides to start antibiotics after sex. Six months later **[name]** makes another appointment and sees Dr Hussein as she has had another three urine infections despite using a variety of over-the-counter treatments and antibiotics after sex. They discuss her infections and **[name]** is keen to start long-term antibiotics as her infections are really impacting home life. The antibiotics work well and **[name]** has less urine infections over the next six months and has another appointment with Dr Morgan to review her long-term antibiotics and whether to continue or stop them.

**What do you think [name] is thinking prior to attending this appointment? [TDF 1** **Knowledge, 6** **Beliefs about Consequences]**

**What information do you think [name] will need to make this decision with her GP? [TDF 1** **Knowledge, 9** **Goals, 10** **Memory, Attention and Decision Processes]**

**How and when would it be best for [name] to receive this information? [TDF 11** **Environmental Context and Resources]**

**How could [name] be supported by her GP to make this decision? [TDF 3** **Social/Professional Role and Identity, 6** **Beliefs about Consequences, 11** **Environmental Context and Resources, 12** **Social Influences]**

**Do you think [name] would want different information at this review compared to when she was deciding whether to start long-term antibiotics? [TDF 1** **Knowledge, 11** **Environmental Context and Resources]**

## 3. 51-year-old – peri menopause

**[name]** is 51 years old. She is a single parent with two young children aged 12 and 16. She thinks she is going through the menopause since for the last 18 months she has been getting hot flushes and her periods are much less frequent. **[name]** has also had three urine infections in the last year. Three days ago, she started noticing it was painful to pass urine, was passing urine more frequently and noticed her urine had started to smell. She thinks she has another urine infection and tries drinking more fluids, but this does not help. **[name]** sees Dr Morgan (GP) who agrees she has a urine infection and prescribes antibiotics. This is the fourth infection **[name]** has had in the last 12 months and she is worried about them. **[name]** thinks they might be related to her going through the menopause. They discuss hygiene and water intake as ways to prevent infections and a follow-up appointment made.

**What do you think is going through [name]’s mind? [TDF 1 Knowledge]**

**Is there anything [name]could be worried about? [TDF 1 Knowledge, 13** **Emotion]**

**What do you think [name] will want to ask the GP at the follow-up appointment? [TDF 6** **Beliefs about Consequences]**

**What information do you think [name] wants about recurrent urine infections? [TDF 1** **Knowledge]**

**How do you think [name] might want to get this information? [TDF 11** **Environmental Context and Resources]**

**[name]** has had a further infection prior to her follow-up appointment despite doing what they discussed at the last appointment. Dr Morgan discusses potential treatments that could reduce the number of infections including using an oestrogen cream every day or taking low dose long-term antibiotics once a day at night. **[name]** is concerned about taking long-term antibiotics as she has heard about the risk of antibiotic not working in the future. **[name]** asks Dr Morgan about any other antibiotic alternative treatments. Dr Morgan says that there are a few other treatments but there is differing information on how good they are at preventing further infections.

**What do you think is going through [name] mind now? [TDF 1** **Knowledge]**

**What information do you think [name] will want to know about taking antibiotics to prevent urine infections when making this decision? [ TDF 1** **Knowledge, 9** **Goals, 10** **Memory, Attention and Decision Processes]**

**What information [name] will they want on these non-antibiotic treatments when making this decision? [ TDF 1** **Knowledge, 9** **Goals, 10** **Memory, Attention and Decision Processes]**

**How might she want the information? [TDF 13** **Emotion]**

**Is there anything you think [name] might be concerned or worried about? [TDF 13** **Emotion]**

**[name]** decides to start the oestrogen cream. Six months later **[name]** makes another appointment and sees Dr Hussein as she has had another three urine infections despite using the cream and using a variety of over-the-counter treatments. They discuss her infections and **[name]** is keen to start long-term antibiotics as her infections are really impacting life. The antibiotics work well and **[name]** has less urine infections over the next six months and has another appointment with Dr Morgan to review her long-term antibiotics and whether to continue or stop them.

**What do you think [name] is thinking prior to attending this appointment? [TDF 1** **Knowledge, 6** **Beliefs about Consequences]**

**What information do you think [name] will need to make this decision with her GP? [TDF 1** **Knowledge, 9** **Goals, 10** **Memory, Attention and Decision Processes]**

**How and when would it be best for [name] to receive this information? [TDF 11** **Environmental Context and Resources]**

**How could [name] be supported by her GP to make this decision? [TDF 3** **Social/Professional Role and Identity, 6, 11** **Environmental Context and Resources, 12]**

**Do you think [name] would want different information at this review compared to when she was deciding whether to start long-term antibiotics? [TDF 1** **Knowledge, 11** **Environmental Context and Resources]**

## 4. 71-year-old – post menopausal

**[name]** is 71 years old. She is retired, married and has three grown up children. Over the last few years [**name]** has been getting more urine infections. In the last year she has had three urine infections and three days ago she started noticing it was painful to pass urine, was passing urine more frequently, she was having to rush to the toilet to pass urine and noticed her urine had started to smell. She thinks she has another urine infection and tries drinking more fluids, but this does not help. **[name]** sees Dr Morgan who agrees she has a urine infection and prescribes three days of antibiotics. **[name]** says this is the fourth infection she has had in the last 12 months, and she is worried about them. **[name]** says the infections have become more common since she went through the menopause and thinks they are more likely to occur after sexual intercourse. They discuss hygiene and water intake as ways to prevent infections and a follow-up appointment made.

**What do you think is going through [name]’s mind? [TDF 1** **Knowledge]**

**Is there anything [name]could be worried about? [TDF 1** **Knowledge, 13** **Emotion]**

**What do you think [name] will want to ask the GP at the follow-up appointment? [TDF 6** **Beliefs about Consequences]**

**What information do you think [name] wants about recurrent urine infections? [TDF 1** **Knowledge]**

**How do you think [name] might want to get this information? [TDF 11** **Environmental Context and Resources]**

**[name]** has had a further infection prior to her follow-up appointment despite doing what they discussed at the last appointment. Dr Morgan discusses potential treatments that could reduce the number of infections including using an oestrogen cream, taking an antibiotic after intercourse or taking low dose long-term antibiotics every day at night. **[name]** is concerned about taking long-term antibiotics as she has heard about the risk of antibiotic not working in the future. **[name]** asks Dr Morgan about any other antibiotic alternative treatments. Dr Morgan says that there are a few other treatments but there is differing information on how good they are at preventing further infections.

**What do you think is going through [name] mind now? [TDF 1** **Knowledge]**

**What information do you think [name] will want to know about taking antibiotics to prevent urine infections when making this decision? [ TDF 1** **Knowledge, 9** **Goals, 10** **Memory, Attention and Decision Processes]**

**What information [name] will they want on these non-antibiotic treatments when making this decision? [ TDF 1** **Knowledge, 9** **Goals, 10** **Memory, Attention and Decision Processes]**

**How might she want the information? [TDF 13** **Emotion]**

**Is there anything you think [name] might be concerned or worried about? [TDF 13** **Emotion]**

**[name]** decides to start antibiotics after sex. Six months later **[name]** makes another appointment and sees Dr Hussein as she has had another three urine infections despite using a variety of over-the-counter treatments and antibiotics after sex. They discuss her infections and **[name]** is keen to start long-term antibiotics as her infections are really impacting home and social life. The long-term antibiotics work well and **[name]** has less urine infections over the next six months and has another appointment with Dr Morgan to review her long-term antibiotics and whether to continue or stop them.

**What do you think [name] is thinking prior to attending this appointment? [TDF 1** **Knowledge, 6** **Beliefs about Consequences]**

**What information do you think [name] will need to make this decision with her GP? [TDF 1** **Knowledge, 9** **Goals, 10** **Memory, Attention and Decision Processes]**

**How and when would it be best for [name] to receive this information? [TDF 11** **Environmental Context and Resources]**

**How could [name] be supported by her GP to make this decision? [TDF 3** **Social/Professional Role and Identity, 6** **Beliefs about Consequences, 11** **Environmental Context and Resources, 12]**

**Do you think [name] would want different information at this review compared to when she was deciding whether to start long-term antibiotics? [TDF 1** **Knowledge, 11** **Environmental Context and Resources]**

## 5. 89-year-old – post menopausal

**[name]** is 89 years old. She lives alone and is a widow since her husband died 5 years ago. She doesn’t have any children. **[name]** has had three urine infections in the last year one of which particularly scared her as she became unsteady on her feet. Three days ago, she started noticing it was painful to pass urine, was passing urine more frequently, she was having to rush to the toilet to pass urine and noticed her urine had started to smell. She thinks she has another urine infection and tries drinking more fluids, but this does not help. **[name]** sees Dr Morgan who agrees she has a urine infection and prescribes three days of antibiotics. **[name]** says this is the fourth infection she has had in the last 12 months, and she is worried about them. **[name]** thinks she has had more infections since going through the menopause and thinks they often occur when she doesn’t drink enough fluids. They discuss hygiene and water intake as ways to prevent infections and a follow-up appointment made.

**What do you think is going through [name]’s mind? [TDF 1** **Knowledge]**

**Is there anything [name]could be worried about? [TDF 1** **Knowledge, 13** **Emotion]**

**What do you think [name] will want to ask the GP at the follow-up appointment? [TDF 6** **Beliefs about Consequences]**

**What information do you think [name] wants about recurrent urine infections? [TDF 1** **Knowledge]**

**How do you think [name] might want to get this information? [TDF 11** **Environmental Context and Resources]**

**[name]** has had a further infection prior to her follow-up appointment despite doing what they discussed at the last appointment. Dr Morgan discusses potential treatments that could reduce the number of infections including using an oestrogen cream every day or taking a low dose long-term antibiotic every day at night. **[name]** is concerned about taking long-term antibiotics as she has heard about the risk of antibiotic not working in the future. **[name]** asks Dr Morgan about any other antibiotic alternative treatments. Dr Morgan says that there are a few other treatments that can be bought in a pharmacy but there is differing information on how good they are at preventing further infections.

**What do you think is going through [name] mind now? [TDF 1** **Knowledge]**

**What information do you think [name] will want to know about taking antibiotics to prevent urine infections when making this decision? [ TDF 1** **Knowledge, 9** **Goals, 10** **Memory, Attention and Decision Processes]**

**What information [name] will they want on these non-antibiotic treatments when making this decision? [ TDF 1** **Knowledge, 9** **Goals, 10** **Memory, Attention and Decision Processes]**

**How might she want the information? [TDF 13** **Emotion]**

**Is there anything you think [name] might be concerned or worried about? [TDF 13** **Emotion]**

**[name]** decides to start the oestrogen cream. Six months later **[name]** makes another appointment and sees Dr Hussein as she has had another three urine infections despite using the cream and using a variety of over-the-counter treatments. They discuss her infections and **[name]** is keen to start long-term antibiotics as her infections are really impacting her life. The antibiotics work well and **[name]** has less urine infections over the next six months and has another appointment with Dr Morgan to review her long-term antibiotics and whether to continue or stop them.

**What do you think [name] is thinking prior to attending this appointment? [TDF 1** **Knowledge, 6** **Beliefs about Consequences]**

**What information do you think [name] will need to make this decision with her GP? [TDF 1** **Knowledge, 9** **Goals, 10** **Memory, Attention and Decision Processes]**

**How and when would it be best for [name] to receive this information? [TDF 11** **Environmental Context and Resources]**

**How could [name] be supported by her GP to make this decision? [TDF 3** **Social/Professional Role and Identity, 6** **Beliefs about Consequences, 11** **Environmental Context and Resources, 12** **Social Influences]**

**Do you think [name] would want different information at this review compared to when she was deciding whether to start long-term antibiotics? [TDF 1** **Knowledge, 11** **Environmental Context and Resources]**
